# Unmet need for induction of labor in Africa: secondary analysis from the 2004 - 2005 WHO Global Maternal and Perinatal Health Survey (A cross-sectional survey)

**DOI:** 10.1186/1471-2458-12-722

**Published:** 2012-08-31

**Authors:** Fawole Bukola, Nafiou Idi, Machoki M’Mimunya, Wolomby-Molondo Jean-Jose, Mugerwa Kidza, Neves Isilda, Amokrane Faouzi, Shah Archana, Souza Joao Paulo, Mathai Matthews, Gulmezoglu Metin

**Affiliations:** 1 Department of Obstetrics & Gynaecology, College of Medicine, University of Ibadan, Ibadan, Nigeria; 2 Department of Obstetrics & Gynaecology, Abdou Moumouni University, Niamey, Niger; 3 Department of Obstetrics & Gynaecology, University of Nairobi, Nairobi, Kenya; 4 Department de Gynaecologie et Obstetrique, Cliniques Universitaire de Kinshasa, Kinshasa, Democratic Republic of Congo; 5 Department of Obstetrics & Gynaecology, Makerere University, Kampala, Uganda; 6 Delegacao Provincial de Saude de Luanda, Luanda, Angola; 7 Ministry of Health, Algiers, Algeria; 8 Department of Health Systems, Policy and Workforce, World Health Organization, Geneva, Switzerland; 9 Department of Reproductive Health and Research, World Health Organization, Geneva, Switzerland; 10 Department of Maternal, Newborn, Child and Adolescent Health, World Health Organization, Geneva, Switzerland

**Keywords:** Induction of labor, Rate, Utilization, Stillbirth, Unmet need, Perinatal death, Indication

## Abstract

**Background:**

Induction of labor is being increasingly used to prevent adverse outcomes in the mother and the newborn.This study assessed the prevalence of induction of labor and determinants of its use in Africa.

**Methods:**

We performed secondary analysis of the WHO Global Survey of Maternal and Newborn Health of 2004 and 2005. The African database was analyzed to determine the use of induction of labor at the country level and indications for induction of labor. The un-met needs for specific obstetric indications and at country level were assessed. Determinants of use of induction of labor were explored with multivariate regression analysis.

**Results:**

A total of 83,437 deliveries were recorded in the 7 participating countries. Average rate of induction was 4.4% with a range of 1.4 – 6.8%. Pre-labor rupture of membranes was the commonest indication for induction of labor. Two groups of women were identified: 2,776 women with indications had induction of labor while 7,996 women although had indications but labor was not induced.

Induction of labor was associated with reduction of stillbirths and perinatal deaths [OR – 0.34; 95% CI (0.27 – 0.43)].

Unmet need for induction of labor ranged between 66.0% and 80.2% across countries. Determinants of having an induction of labor were place of residence, duration of schooling, type of health facility and level of antenatal care.

**Conclusion:**

Utilization of induction of labor in health facilities in Africa is very low. Improvements in social and health infrastructure are required to reverse the high unmet need for induction of labor.

## Background

Induction of labor with the goal of achieving vaginal delivery prior to spontaneous onset of labor is recommended when the benefits of delivery outweigh the risks of continuing the pregnancy [1]. Major indications for induction of labor include maternal, fetal, social or a combination of these factors; these indications may also ‘either be evident or anticipated’ [2].

Rates of induction of labor vary from region to region. In the United States of America and the United Kingdom, about 20% of all deliveries are by induction of labor [1,3,4] while 11.4% was reported for Latin America [5]. Rates of induction of labor are low in the African region. Only 3% of women had induction of labor in a specialist unit in Nigeria [6].

Induction of labor is directly relevant to the health related millennium development goals (MDGs). It has potentials for preventing maternal complications and improving pregnancy outcome. Beyond 41 weeks gestation, the number of routine induction of labor needed to prevent 1 fetal or neonatal death decreases constantly [7]. An increased rate of induction of labor for post-term pregnancies over a 15-year period was associated with decreased stillbirth rates in Canada [8]. Higher rates of induction of labor may also contribute to lowering caesarean section rates without increasing other adverse pregnancy outcomes [9]. In otherwise uncomplicated singleton pregnancies, meta-analysis of randomized controlled trials showed that a policy of induction of labor at 41 weeks reduces caesarean section rates without adverse perinatal outcomes [10]. Minimizing caesarean section rates without increasing other adverse pregnancy outcomes is a priority consideration in low income countries where available resources need to be judiciously utilized.

Africa has the highest maternal mortality [11] as well as the highest stillbirth and perinatal mortality rates [12]. Efforts aimed at achieving the health related MDGs should focus on increasing access to effective interventions and on improving quality of health care [11]. In this context, utilization of induction of labor within the African sub-region deserves closer scrutiny.

We analyzed the African data set in the WHO Global Maternal and Perinatal Health Survey of 2004 – 2005 to assess practice of induction of labor within African countries and its implications for maternal and newborn health. Specifically, we aimed to determine the proportion of pregnant women who could have benefitted from the intervention but did not receive it and also compare pregnancy outcomes for women who had induction of labor and those who were eligible but missed the opportunity.

## Methods

The WHO Global Survey of Maternal and Perinatal Health was a cross-sectional survey implemented in 373 health facilities selected by stratified multi-stage cluster sampling design in 24 countries in Africa, Asia and Latin America between 2004 and 2005. This study is a secondary analysis of African data from the database. Detailed methodological considerations for the survey have been previously published [13,14]. Within Africa, 131 health facilities participated in the survey. Participating countries were selected from the 14 sub-regions of the WHO classified according to under-five and adult mortality rates which served as proxy for the burden of maternal and perinatal mortality [15]. In each sub-region, four countries were selected with probability proportional to their population size. In sub-regions with less than four countries, all countries within that sub-region were included. At the country level, the capital city was always selected. Two other provinces were further selected randomly among the remaining provinces. Within each province and the capital city, seven health facilities were randomly selected among a sample of health facilities that reported at least 1000 deliveries in the year prior to the implementation of the survey. If there were fewer than seven eligible health facilities in the capital city or the provinces, then all available health facilities were selected. Eligible health facilities were identified from an up to date list of all health facilities and their annual delivery rates in the selected provinces previously prepared by the Country coordinators in collaboration with WHO country offices and Ministry of Health.

Data collection was for 2 months in health facilities with 6,000 or more deliveries per annum and 3 months in health facilities with less than 6,000 deliveries per annum. All women who delivered in the health facilities during the study period and their newborns were included in the survey. Data were obtained at the individual patient and the health facility levels. Facility level data included type of facility (primary without surgical facilities, secondary, or tertiary with surgical facilities), location and availability of relevant laboratory tests, anesthesiology resources and resources for intrapartum care including emergency obstetric care. Individual level data were abstracted from patient’s medical records onto a 54-item data collection form. The woman’s socio-demographic characteristics, obstetric risk factors, details of pregnancy and antenatal care, mode of delivery and maternal and newborn outcomes up to hospital discharge or up to a maximum stay of seven days were documented by a midwife. Monitoring of infants within the health facility was discontinued at discharge or on the 7^th^ day postpartum whichever was sooner. Discrepancies and incomplete data in medical records were resolved in consultation with the attending obstetric staff prior to the patient’s discharge. Data collection was also facilitated by a manual of operations to ensure uniformity of data. Ethical approval for the study was obtained from WHO’s Scientific and Ethical Review Group and Ethics Review Committee and from the participating health facilities. Consent was not obtained from the mother because there was no direct contact with the individual participant. The data collection instruments were pre-tested prior to commencement of the study.

The survey was implemented at the facility level by a trained team of hospital coordinator and a midwife. Online data entry onto a global database was done at the country level by a trained data clerk. The global database was managed by MedSciNet AB (Stockholm) in collaboration with the WHO coordinating team.

### Statistical analysis

Statistical analysis was performed with SPSS software version 17.0. Maternal age, total number of years attended school, total number of antenatal visits, maternal status at discharge, status at birth, apgar score at 5 min, birth weight and newborn status at discharge were re-classified to facilitate analysis. We determined the rates of induction of labor at the continent and country levels and compared the rates of induction with the rates of spontaneous onset of labor and elective caesarean delivery. Indications for induction of labor by country were also determined. We examined the mode of delivery following induction of labor and maternal and perinatal mortality associated with the procedure. To assess the effects of induction of labor as an obstetric intervention in term pregnancies, we identified women with term pregnancies who had an indication for induction of labor. Two sub-sets of women were further identified: women with an indication for induction of labor, who had the procedure (Induced) and women who had an indication for induction of labor but did not receive induction of labor (Not induced).

Women were eligible for this sub-group analysis if pregnancy was ≥ 37 weeks and associated with any of the following complications: pregnancy induced hypertension, chronic hypertension, pre-eclampsia, cardiac/renal diseases, chronic respiratory conditions, uterine height low for gestational age, diabetes mellitus and post-term pregnancy. Women with intra-uterine fetal death at ≥ 28 weeks gestation who had induction of labor were categorized as not receiving induction of labor. Women with haemoglobinopathies at ≥ 41 weeks of gestation who met the eligibility criteria were included in the analysis. Women with the following conditions were excluded from the sub-group analysis: women with history of vesico-vaginal fistula/rectovaginal fistula, previous surgery on uterus and cervix, caesarean section at last pregnancy and women delivered by elective caesarean section in the index pregnancy. Women with live fetuses were excluded when presentation was other than cephalic. Cases of eclampsia were also excluded from the analysis. The time interval between pre-labor rupture of membranes and spontaneous onset of labor or induction of labor was not captured in the database. These women were therefore excluded from this analysis. Two sub-groups of women were thus generated: Women with a live fetus who had induction of labor at term (Induced); and women with term pregnancies who had indications for induction of labor but did not have induction of labor and women who had intra-uterine fetal death at ≥ 28 weeks (Not induced). The two groups were compared with respect to maternal and perinatal outcomes. Unmet need for induction of labor for the selected complications of pregnancy was derived from the ratio of women who had indications for induction of labor but did not receive it and the total number of all women who had such indications multiplied by 100.

Associations were tested using chi square or Fisher’s exact test as appropriate; the level of statistical significance was set at p < 0.05. We employed multivariate logistic regression analysis with analysis of variance to assess independent determinants of women having an induction of labor.

## Results

**Figure 1:**
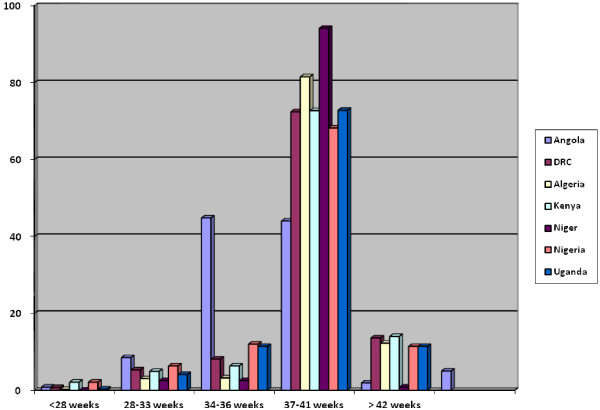
Percentage distribution of gestational age at induction of labor by country.

The total number of deliveries for all countries reported during the survey was 83,437. There were 81,941 singleton births, 1,437 twin deliveries, 38 triplets and one set of quadruplets and quintuplets each; number of neonates was not documented for 19 births. Congenital malformations were reported in 1,570 newborns. Total live births were 80,297 while fresh stillbirths and macerated stillbirths were 1,855 and 1,199 respectively; 950 early neonatal deaths occurred prior to mother’s discharge from the hospital. Almost 70% of the reported fetal deaths occurred beyond 34 weeks. The mode of onset of labor and the rates of induction of labor by country are shown in Table 1. Rates of induction of labor were variable from country to country, ranging between 1.4% in Niger to 6.8% in Algeria. Overall rate of induction for the African region was 4.4%. Most inductions were performed between 37 and 41 weeks of gestation (Figure 1). However, in Angola, there was an even distribution of induction of labor between 34 – 36 weeks (44.8%) and 37 – 41 weeks (44.0%). Although there were 3,700 positive responses to induction of labor, however, an indication for induction of labor was reported for 4,736 women. Indication for induction of labor according to gestational age is shown in Table 2. Pre-labor rupture of membranes was the commonest indication for induction of labor. Term pre-labor rupture of membranes (i.e. gestational age ≥ 34 weeks) accounted for 95% of these cases. Using the pre-specified criteria for selected complications of pregnancy to identify women who had an indication for induction of labor, 2,776 women had induction of labor while 7,996 women with indications for induction of labor did not receive the intervention. The frequency of stillbirths and sperinatal deaths in the two groups is shown in Table 3. There was an apparent reduction of stillbirths and perinatal deaths among women who had induction of labor compared with women who did not. This difference was most obvious among women with selected medical complications of pregnancy where doubling of the risk for both stillbirths and perinatal deaths was noted among women who did not have induction of labor.

**Table 1:** Onset of labor by country

**Country/Region**	**Spontaneous onset of labor**		**Induction of labor**		**No labor**		**Total**
	**N**	**%**	**N**	**%**	**N**	**%**	
Angola	6003	93.5	322	5.0	97	1.5	6422
^*^DRC	8404	93.3	462	5.1	142	1.6	9008
Algeria	13795	86.8	1073	6.8	1019	6.4	15887
Kenya	18813	92.6	792	3.9	720	3.5	20325
Niger	8279	98.2	118	1.4	38	0.5	8435
Nigeria	8249	89.8	577	6.3	364	4.0	9190
Uganda	13422	95.2	356	2.5	323	2.3	14101
Africa	76965	92.3	3700	4.4	2703	3.2	83368

^*^DRC - Democratic Republic of Congo.

**Table 2:** Indications for induction of labor by gestational age (weeks)

**Indication**	**< 28**		**28-33**		**34-36**		**37-41**		**≥ 42**		**Overall %**
	**N**	**%**	**N**	**%**	**N**	**%**	**N**	**%**	**N**	**%**	
Fetal death (N - 291)	10	3.4	85	29.2	62	21.3	121	41.6	13	4.5	6.2
IUGR (N - 77)	2	2.6	9	11.7	16	20.8	48	62.3	2	2.6	1.6
Fetal distress (N-675)	1	0.1	10	1.5	61	9.0	580	85.9	23	3.4	14.3
Multiple pregnancy (N - 100)	2	2.0	1	1.0	11	11.0	84	84.0	2	2.0	2.1
PROM (N - 981)	9	0.9	40	4.1	154	15.7	750	76.5	28	2.9	20.8
Chorio-amnionitis (N - 55)	1	1.8	4	7.3	13	23.6	33	60.0	4	7.3	1.2
Vaginal bleeding (N - 80)	1	1.3	10	12.5	9	11.3	55	68.8	5	6.3	1.7
Pre-eclampsia/eclampsia (N - 291)	7	2.4	14	4.8	29	10.0	232	79.7	9	3.1	6.2
Post term ≥ 42 weeks (N - 540)	-	-	-	-	-	-	-	-	540	100.0	11.5
Elective induction (N - 333)	-	-	4	1.2	10	3.0	292	87.7	27	8.1	7.1
Maternal request (N - 67)	1	1.5	-	-	3	4.5	63	94.0	-	-	1.4
Any other pregnancy complication (N - 703)	7	1.0	23	3.3	19	2.7	636	90.5	18	2.6	14.9
Any other maternal medical complication (N - 523)	1	0.2	10	1.9	22	4.2	477	91.2	13	2.5	11.1

Missing values - 20.

**Table 3:** frequency of stillbirths and perinatal deaths in induced and not induced groups

**Category**	**N**	**Stillbirths**		**Perinatal deaths**	
		**N**	**%**	**N**	**%**
**Induced**					
Selected medical complications induced at ≥ 37 weeks	2776	66	2.4	88	3.2
**Not Induced**					
Selected medical complications at ≥ 37 weeks with spontaneous labor onset	2384	133	5.6	164	6.9
Pregnancies > 41 weeks with spontaneous labor onset	3397	59	1.7	77	2.3
Pregnancies with no known medical complications at ≥ 42 weeks with spontaneous labor onset	1838	62	2.2	83	4.5
Medical complications and IUFD at ≥ 28 weeks	109	109	-	109	-
Pregnancies with medical complications between 28 and 36 weeks ending as fresh or macerated stillbirths	268	268	-	268	-
**Total (Not Induced)**	**7996**	**626**	**7.8**	**701**	**8.8**

IUFD – Intra-uterine fetal death.

Thus, perinatal mortality rate among women in the Induced group was 31.7 per 1,000 deliveries while perinatal mortality rate among the Not Induced group was 87.7 per 1,000 deliveries. These differences were statistically significant [OR – 0.34 95% CI (0.27 – 0.43)], implying that induction of labor results in about 66% reduction of perinatal deaths.

For the selected complications of pregnancy we determined the proportion of women who could not benefit from induction of labor (i.e. the unmet need for induction of labor) and its effect on the newborn (Table 4). For all the complications shown in Table 4, the vast majority of women had a huge unmet need for induction of labor. Apparently for most of these conditions, induction of labor was associated with significant reduction in perinatal deaths e.g. induction for pre-eclampsia, severe anemia and other medical conditions were associated with 78%, 87%, 83% reduction in perinatal deaths respectively. Induction of labor for post-term pregnancy, diabetes and IUGR had no significant impact on perinatal deaths. Unmet need for induction of labor at the country level and perinatal death is shown in Table 5. Angola, where the induction rates between 34 and 36 weeks surpasses the induction rates at term, had the lowest unmet need for induction of labor (66%); unmet need in most countries was above 70%. Thus, unmet need for induction of labor was relatively high in all countries. However, the intervention was significantly associated with reduction of perinatal deaths in all countries ranging between 53 and 67%.

**Table 4:** Unmet need for induction of labor and perinatal deaths

**Pregnancy complication**	**Not Induced**	**Induced**	**Unmet Need for Induction (%)**	**Perinatal deaths**				**OR (95% CI)**
				**Not Induced**		**Induced**		
				**N**	**%**	**N**	**%**	
Pregnancy induced hypertension (N - 1371)	1351	220	86.0	161	11.9	6	2.7	0.21 (0.09 – 0.47)
Chronic hypertension (N - 263)	241	22	91.6	39	16.2	1	4.6	0.25 (0.03 – 1.89)
Pre-eclampsia (N - 974)	812	162	83.4	122	15.0	6	3.7	0.22 (0.09 – 0.50)
Cardiac/Renal disease (N - 159)	144	15	90.6	11	7.6	0	0.0	-
Chronic respiratory conditions (N - 307)	294	13	95.8	16	5.4	0	0.0	-
IUGR (N - 345)	315	30	91.3	53	16.8	3	10.0	0.55 (0.16 – 1.88)
Diabetes (N - 216)	186	30	86.1	25	13.4	0	0.0	-
Malaria (N - 915)	763	152	83.4	175	22.9	5	3.3	0.11 (0.05 – 0.28)
Sickle cell disease (N - 3)	-	3	-	-	-	0	-	-
Severe anemia (N- 131)	98	33	74.8	33	33.7	2	6.1	0.13 (0.03 – 0.56)
Vaginal bleeding in second half of pregnancy (N - 148)	119	29	80.4	79	66.4	6	20.7	0.13 (0.05 – 0.35)
Pyelonphritis/UTI (N-484)	363	121	75.0	40	11.0	2	1.7	0.14 (0.03 – 0.57)
Other medical conditions (N - 523)	385	138	73.6	58	15.1	4	2.9	0.17 (0.06 – 0.47)
Post term pregnancy (N - 2232)	1845	387	82.7	85	4.6	16	4.1	0.89 (0.50 – 1.58)

IUGR – Intra-uterine growth restriction.

**Table 5:** Unmet need for induction of labor and perinatal deaths at country level

**Country**	**Not Induced**	**Induced**	**Unmet need for induction of labor (%)**	**Perinatal mortality**				**OR (95% CI)**
				**Not Induced**		**Induced**		
				**N**	**%**	**N**	**%**	
Angola (N - 332)	219	113	66.0	30	13.7	4	3.5	0.23 (0.07 – 0.71)
DRC (N - 1539)	1204	335	78.2	88	7.3	12	3.6	0.47 (0.24 – 0.90)
Algeria (N - 3323)	2383	940	71.7	131	5.5	14	1.5	0.26 (0.14 – 0.46)
Kenya (N - 2548)	1919	629	75.3	131	6.8	12	1.9	0.27 (0.14 – 0.50)
Niger (N - 463)	356	107	76.9	50	14.1	5	4.7	0.30 (0.10 – 0.81)
Nigeria (N - 1372)	957	415	69.8	155	16.2	31	7.5	0.42 (0.27 – 0.64)
Uganda (N - 1195)	958	237	80.2	113	11.8	10	4.2	0.33 (0.16 – 0.66)

We assessed the effects of induction of labor on some other maternal and newborn outcomes (Table 6). The intervention also had significant impacts on some other newborn outcomes. Women who had induction of labor had 71% reduction of risk of having fresh or macerated stillbirths. However, the newborn was 25% more likely to be admitted into the intensive care unit following induction of labor.

**Table 6:** Other maternal and newborn outcomes

	**Not induced**		**Induced**		**OR (95% CI)**
	**N**	** %**	**N**	** %**	
**Maternal**					
Mode of delivery					
Vaginal delivery	6668	83.5	2283	82.4	1.08 (0.96 – 1.21)
Emergency CS	1317	16.5	487	17.6	
**Hysterectomy**					
No	7989	99.9	2771	99.8	2.0 (0.83 – 10.00)
Yes	5	0.1	5	0.2	
**Maternal status at discharge**					
Alive	7944	99.3	2761	99.5	0.83 (0.47 – 1.48)
Dead	52	0.7	15	0.5	
**Newborn Status at birth**					
Alive	7370	92.2	2709	97.6	0.29 (0.22 – 0.37)
Fresh/macerated s till birth	626	7.8	66	2.4	
**Admission into intensive care**					
No	6570	89.2	2351	86.8	1.25 (1.09 – 1.43)
Yes	796	10.8	357	13.2	

For the mother, induction of labor was not significantly associated with increased risk of emergency caesarean section, hysterectomy or maternal death (Table 6).

The woman’s age, parity, duration of schooling and antenatal care were associated with induction of labor (Table 7). Maternal age < 20 years, < 4 antenatal visits and low level of education were associated with reduced rates of induction of labor while urban location, care at a tertiary health facility or teaching facility were associated with higher rates of induction of labor (Table 8).

**Table 7:** Maternal characteristics and induction of labor

	** Not induced**		** Induced**		**Significance**
	**N**	** %**	**N**	** %**	
**Age group (years)**					
<20	1235	80.4	298	19.4	*χ*^2^ – 60.4; p = 0.000 (S)
21 – 25	2026	76.2	634	23.8	
26 – 30	2229	72.2	858	27.8	
31 – 35	1467	70.6	612	29.4	
36 – 40	824	72.8	308	27.2	
>40	185	76.4	57	23.6	
**Duration of schooling (years)**					
0	809	78.4	223	21.6	*χ*^2^ – 137.1 p = 0.000
1 – 6	1173	75.4	382	24.6	
7 – 12	4208	76.3	1306	23.7	
13 – 18	1165	63.5	671	36.5	
>18	31	62.0	19	38.0	
**Parity**					
0	2956	71.1	1203	28.9	*χ*^2^ – 38.3 p = 0.000
1 – 2	2847	75.6	920	24.4	
3 – 4	1320	77.5	384	22.5	
≥5	797	76.9	240	23.1	
**Number of antenatal visits**					
0	349	76.5	107	23.5	*χ*^2^ – 180.8 p = 0.000
1 - 3	3628	80.8	863	19.2	
≥4	3186	68.6	1460	31.4	

**Table 8:** Health facility characteristics and induction of labor

	**Not Induced**		** Induced**		**Significance**
	**N**	** %**	**N**	** %**	
**Location**					
Urban	6137	71.9	2403	28.1	*χ*^2^ – 124.2 p = 0.000
Peri-urban	570	79.4	148	20.6	
Rural	1245	85.0	219	15.0	
**Teaching facility?**					
No	3612	79.7	918	20.3	*χ*^2^ – 120.2 p = 0.000
Yes	4265	70.3	1799	29.7	
**Level of health facility**					
Primary	823	81.4	188	18.6	*χ*^2^ – 90.9 p = 0.000
Secondary	4411	74.4	1516	25.6	
Tertiary	2086	69.3	926	30.7	
Other	582	82.4	124	17.6	
**Are most patients charged fees?**					
No	2618	70.2	1113	29.8	p = 0.000; OR – 0.73; 95% CI (0.66 – 0.80)
Yes	5377	76.4	1663	23.6	
**Does caesarean section represent economic benefit to attending staff?**					
No	6161	74.2	2142	25.8	p = 0.000; OR – 1.27; 95% CI (1.13 – 1.42)
Yes	1248	69.4	549	30.6	
**Is caesarean section more expensive than normal delivery?**					
No	2007	69.9	863	30.1	p = 0.000; OR – 0.79; 95% CI (0.72 – 0.87)
Yes	4535	74.6	1544	25.4	

In order to determine which characteristics in either the mother or the health facility predicted the likelihood of having induction of labor, variables with significant impact on maternal and newborn outcomes were entered into a multivariate logistic regression model. All maternal and health facility characteristics that were significantly associated with induction of labor were shown to independently predict the likelihood of the mother having induction of labor (Table 9). When these characteristics were further subjected to stepwise regression, the relative importance of these characteristics in predicting induction of labor was in the following order: location of the woman was the strongest predictor followed by duration of schooling, level of the health facility, whether the facility was teaching or non-teaching and antenatal care. Payment for delivery, higher cost of caesarean section and caesarean section being of economic benefit to attending staff were weaker predictors of a woman having induction of labor.

**Table 9:** Independent predictors of induction of labor

**Characteristic**	**p value**	**OR**	**95% CI**
**Location**	**0.000**		
Urban	-	1	-
Peri-urban	0.004	0.70	0.55 – 0.89
Rural	0.000	0.38	0.31 – 0.46
**Maternal age**	**0.020**		
<20	0.004	0.73	0.60 – 0.91
21 – 25	0.016	0.83	0.71 – 0.97
26 – 30	-	1	-
31 – 35	0.50	1.06	0.90 – 1.24
36 – 40	0.73	0.96	0.78 – 1.19
>40	0.55	0.88	0.59 – 1.32
**Schooling duration**	**0.001**		
0	0.014	0.75	0.59 – 0.94
1 – 6	0.031	0.81	0.66 – 0.98
7 – 12	0.000	0.72	0.62 – 0.84
13 – 18	0.44	0.74	0.34– 1.60
>18	-	1	-
**Parity**	**0.001**		
0	0.01	1.25	1.09 – 1.44
1 – 2	-	1	-
3 – 4	0.21	0.89	0.75 – 1.07
≥5	0.79	1.03	0.82 – 1.30
**Level of facility**	**0.000**		
Primary	0.037	0.78	0.61 – 0.99
Secondary	0.000	1.40	1.20 – 1.62
Tertiary	-	1	-
Other	0.000	0.36	0.28 – 0.47
**Type of facility**			
Non-teaching	0.000	0.53	0.46 – 0.61
Teaching	-	1	-
**Antenatal care**	**0.000**		
0	0.046	0.76	0.58 – 0.99
1 – 3	0.000	0.66	0.59 – 0.75
≥4	-	1	-
**Delivery charges**			
No	0.000	1.98	1.68 – 2.35
Yes	-	1	-
**Is caesarean section more expensive?**			
No	0.000	0.72	0.60 – 0.86
Yes	-	1	-
**Is caesarean section of economic benefit to attending staff?**			
No	0.000	1.38	1.18 – 1.62
Yes	-	1	-

Thus, compared with urban dwellers, women in peri-urban or rural locations were significantly less likely to receive induction of labor; compared with women in tertiary health facilities, women in secondary health facilities were significantly more likely to receive induction of labor while those in primary health facilities were significantly less likely to receive it; women in non-teaching facilities were significantly less likely to have induction of labor and when women received no antenatal care or made less than 4 antenatal visits, they were significantly less likely to have induction of labor compared with women who had 4 or more visits.

## Discussion

Induction of labor is an obstetric intervention usually employed to prevent adverse pregnancy outcomes. Given the increasing attention to reducing perinatal morbidity and mortality, rates of induction of labor have continued to rise over the past few decades. For example, in the United States, the proportion of live births delivered by induction of labor increased from 9.0% in 1989 to 19.2% in 1998 [3].

Induction rate of 4.4% for the Africa region reported in this study confirms that health facilities in Africa truly have the lowest rates of induction of labor [16]. These low rates of induction of labor in Africa closely reflect the very high perinatal mortality rate of 56 per 1000 live births for the region according to WHO estimates [12], the highest in all the world regions. Whereas the induction rates in Africa are about one-fifth to a quarter of the rates for more developed regions, its perinatal mortality rate is 7 – 8 fold higher. Data from this study showed almost 50% reductions in stillbirths and perinatal deaths when induction of labor was employed in the presence of medical complications in term pregnancies. Specifically for hypertensive disorders of pregnancy, induction of labor was significantly associated with more than 75% reduction in perinatal mortality.

Induction of labor has also been shown to improve maternal outcomes. Koopmans et al. [17] demonstrated 29% reduction in poor maternal outcome among women with gestational hypertension or pre-eclampsia who had induction of labor compared with expectant management. In this study, proxies for poor maternal outcome namely hysterectomy or maternal death were not significantly different for women who had an induction compared with those who did not.

Pre-labor rupture of membranes was the commonest indication for induction of labor. It was also the commonest indication for induction in Latin America [5]. This contrasts with the experience in the United States where hypertensive disorders of pregnancy constitute commonest indications for induction of labor [3]. Intra-uterine fetal death accounting for almost 7% of all inductions in the African obstetric population represented avoidable perinatal losses. The equivalent figure in Latin America was 2.8% [5]. Induction of labor on account of intra-uterine death will become a thing of the past when induction as an obstetric intervention is employed more frequently.

A major strength of this study was the prospective collection of data. The study was also simultaneously implemented in all participating countries. However, an important limitation was in connection with follow up of the infants. Mothers and infants who were discharged home prior to the seventh day were not followed up further. Some perinatal deaths may have occurred post-discharge from the hospital. Despite this weakness, we consider our data to be robust and representative of contemporary practice within the African region.

Our findings reveal huge unmet needs for induction of labor for all pregnancy conditions and at the country level. This unmet need is a proxy for poor quality obstetric care as well as inadequate access to reproductive health care. These assumptions are supported by the determinants of having an induction of labor. Place of residence shown to be a determinant for induction of labor is a reflection of inequitable distribution of health infrastructure and health personnel in Africa. Duration of schooling also highlights the importance of education as a determinant of reproductive health outcomes. Measures to address the huge unmet need for induction of labor should include the following: integrated rural development and equitable distribution of health infrastructure and manpower; widespread dissemination and adoption of guidelines for obstetric care; strategies and policies to promote female education and strengthening of the referral system. These interventions constitute some of the basic requirements for achieving Millennium Development Goals (MDGs) which have universal access to reproductive health care as one of the key objectives.

Induction of labor however is not without its own risks. Although not statistically significant, women who had an induction had a slightly excess risk of being delivered by emergency caesarean section. The baby also was significantly more likely to be admitted into the intensive care unit. Induction of labor requires close monitoring and availability of blood, surgical, anaesthetic and neonatal facilities. Induction of labor in primary health facilities is thus a major source of concern considering that they may lack these basic emergency obstetric care requirements. Therefore efforts to promote increased utilization of induction of labor in Africa must also give serious consideration to safety issues.

## Conclusion

Induction of labour as an intervention to prevent adverse perinatal outcomes is under-utilized in Africa. This unmet need for induction of labour is a reflection of poor quality of care contributing to the high perinatal rates in the region. Pragmatic measures are required to increase equitable utilization of induction of labour in Africa.

## Competing interests

The Authors declare that they have no competing interests.

## Authors’ contributions

FB - Participated in the design and coordination of the study and helped to draft the manuscript. NI - Participated in the design and coordination of the study. MM - Participated in the design and coordination of the study. WJ - Participated in the design and coordination of the study. MK - Participated in the design and coordination of the study. NI - Participated in the design and coordination of the study. AF - Participated in the design and coordination of the study. SA - Participated in the design and coordination of the study. SJ - Participated in the design and coordination of the study and helped to draft the manuscript. GAM - Participated in the design and coordination of the study and helped to draft the manuscript. All Authors read and approved the final version of the manuscript.

## Pre-publication history

The pre-publication history for this paper can be accessed here:

http://www.biomedcentral.com/1471-2458/12/722/prepub
